# Multitargeting Pt(IV) Derivatives of Cisplatin or Oxaliplatin Inhibit Tumor Growth in Mice without Inducing Neuropathic Pain

**DOI:** 10.1021/acs.jmedchem.4c02263

**Published:** 2025-01-08

**Authors:** Tomer Babu, Ram Pravin Kumar Muthuramalingam, Wei Heng Chng, Jia Ning Nicolette Yau, Sourav Acharya, Nurit Engelmayer, Rachel Feldman-Goriachnik, Shaya Lev, Giorgia Pastorin, Alexander Binshtok, Menachem Hanani, Dan Gibson

**Affiliations:** † Institute for Drug Research, School of Pharmacy, The Hebrew University of Jerusalem, Jerusalem 9112102, Israel; ‡ Department of Pharmacy and Pharmaceutical Sciences, Faculty of Science, National University of Singapore, Singapore 117544, Singapore; § Department of Medical Neurobiology, Institute for Medical Research Israel-Canada, The Hebrew University-Hadassah School of Medicine, Jerusalem 91120, Israel; ∥ The Edmond and Lily Safra Center for Brain Sciences, The Hebrew University of Jerusalem, Jerusalem 9190401, Israel; ⊥ Laboratory of Experimental Surgery, Hadassah-Hebrew University Medical Center, Mount Scopus, Jerusalem 91240, Israel; # Faculty of Medicine, Hebrew University of Jerusalem, Jerusalem 91120, Israel

## Abstract

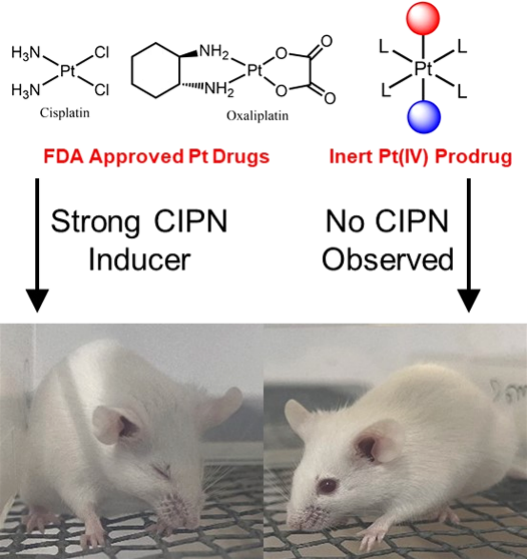

Cisplatin and oxaliplatin are Pt(II) anticancer agents that are used to treat several cancers, usually in combination with other drugs. Their efficacy is diminished by dose-limiting peripheral neuropathy (PN) that affects ∼70% of patients. PN is caused by selective accumulation of the platinum drugs in the dorsal root ganglia (DRG), which overexpress transporters for cisplatin and oxaliplatin. To date, no drug is recommended for the prevention of PN. We report that Pt(IV) prodrugs of cisplatin or oxaliplatin do not induce neuropathic pain in mice, likely due to the lower accumulation of platinum in the DRG compared with Pt(II) drugs. Moreover, the multitargeting prodrug that combines cisplatin with paclitaxel, both strong inducers of PN, efficiently inhibited tumor growth *in vivo* without inducing neuropathic pain. The high antitumor efficacy of Pt(IV) prodrugs and their micellar counterparts and the low level of neuropathic pain associated with them make them ideal candidates for clinical use in cancer therapy.

## Introduction

Even in the age of targeted drugs and immunotherapy, chemotherapy, which is based on antineoplastic drugs, is still widely used as it extends progression-free survival and overall survival and, for certain cancers, is an essential first-line treatment.^1,2^ Chemotherapeutic drugs suffer from inherent and acquired resistance, and treatments are often accompanied by severe side effects.^3^ One of the main side effects associated with most of these drugs is chemotherapy-induced peripheral neuropathy (CIPN), which is dose-limiting and thereby affects the clinical regimens, the outcome of the treatment, and the quality of life of the patients.^4,5^ CIPN is characterized by symptoms of neuropathic pain such as hyperalgesia and allodynia—increased sensitivity to innocuous and noxious heat, cold and mechanical stimuli and paresthesia, pain in the extremities, sensations of numbness, tingling, burning pain, vibration sensation, etc.^6−8^ Platinum drugs (cisplatin or oxaliplatin) or taxanes (paclitaxel or docetaxel) are associated with increased risk of CIPN,^9^ which can appear in the form of acute and/or chronic peripheral neuropathy. Despite the proven efficacy of platinum drugs in treating various cancers, severe side effects accompany the oncological treatments, including peripheral neuropathy.^10^ Oxaliplatin, which is used to treat metastatic colon cancer,^11^ elicits symptomatic neurotoxicity in more than 70% of the patients,^12,13^ often leading to discontinuation of the treatment.^14,15^

Therapeutic interventions for CIPN are very limited since no drug is recommended for CIPN prevention; currently, only duloxetine is recommended for CIPN treatment (moderate recommendation by American Society of Clinical Oncology (ASCO)).^16−18^ Given the propensity of platinum drugs to induce both acute and chronic CIPN, investigating new approaches, which could minimize the neuropathic pain associated with these drugs, while retaining their anticancer activity, is of crucial importance, as platinum drugs are used in at least 50% of all chemotherapeutic regimens.^19^

**Figure 1:**
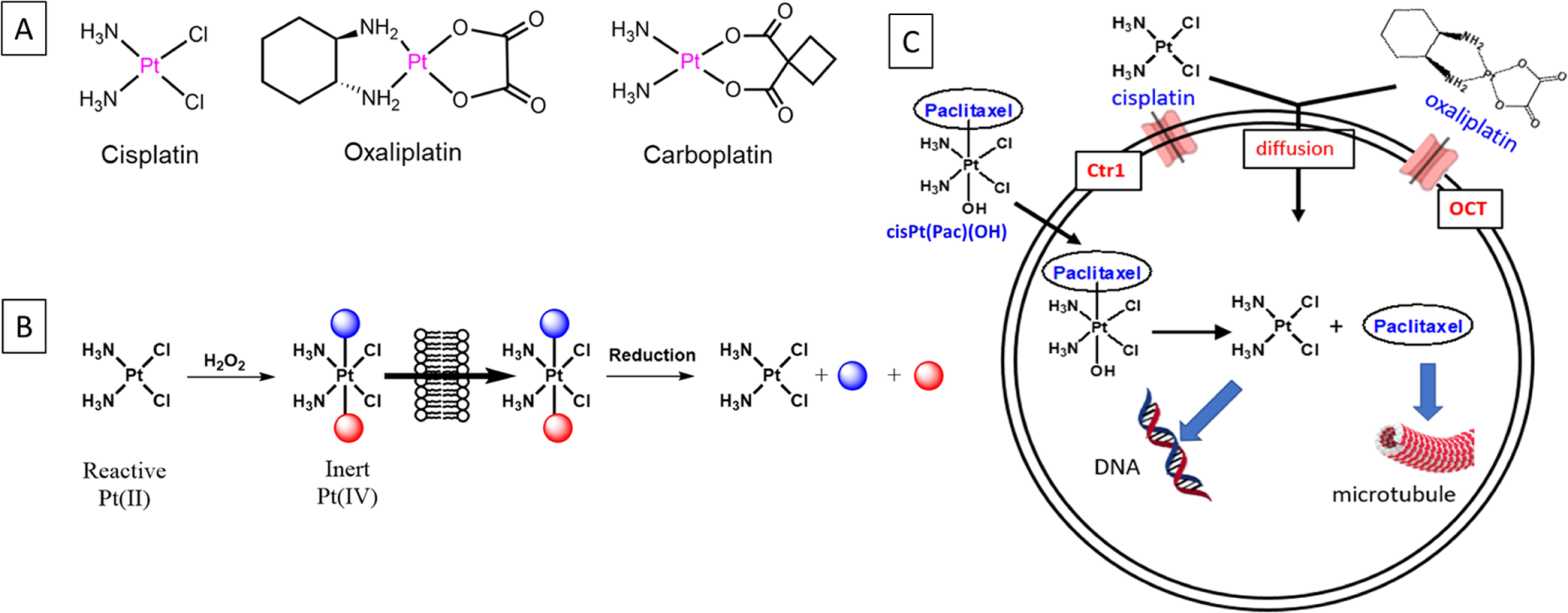
(A) 4-Coordinate Pt(II) complexes approved by the FDA as anticancer drugs. (B) Stable 6-coordinate Pt(IV) complex enters the cell by passive diffusion and is reduced, releasing the Pt(II) drug and the two axial ligands. (C) Cisplatin and oxaliplatin enter the cell by diffusion and via transporters and the dual-targeting cisPt(Pac) (OH) enters the cell by passive diffusion, is reduced, and releases cisplatin and paclitaxel that have different pharmacological targets.

Four-coordinate square planar Pt(II) drugs such as cisplatin or oxaliplatin (Figure 1A) enter cancer cells by passive diffusion as well as by the copper transporter Ctr1 (for cisplatin)^20^ and the organic cation transporters (OCT1/2) (for oxaliplatin).^21,22^ Inside the cells they bind to nuclear DNA, distorting its structure and triggering apoptosis.^23^

One approach to overcome the drawbacks of Pt(II) drugs is to utilize Pt(IV) complexes as prodrugs that deliver the Pt(II) drugs to the tumor. Pt(IV) prodrugs are inert, six-coordinate complexes, which are usually obtained by H_2_O_2_ oxidation of the four-coordinate square planar Pt(II) drugs, resulting in the addition of two hydroxido axial ligands that can be chemically modified. Upon entering cancer cells, the Pt(IV) complex is reduced by intracellular reducing agents, resulting in the breaking of the bonds between the platinum and the axial ligands, thereby regenerating the original Pt(II) drug plus the two additional axial ligands (Figure 1B).^24^ Platinum drugs are in wide clinical use, primarily in combination with other drugs in order to overcome resistance.

We and others have demonstrated that multitargeting Pt(IV) prodrugs, formed by conjugating bioactive axial ligands such as enzyme inhibitors or FDA-approved drugs to the axial positions, can simultaneously deliver to the tumor two or three bioactive moieties that act synergistically to kill cancer cells and overcome drug resistance.^25^ These multitargeting prodrugs have demonstrated good *in vivo* activity but, most importantly, they were also significantly less toxic than the individual drugs or their coadministration.^26,27^

Platinum-induced peripheral neuropathy (PIPN) occurs due to the selective accumulation of the drugs in dorsal root ganglia (DRG), facilitated by the overexpression of the platinum transporters Ctr1 and organic cation transporters in ganglia.^28−30^ Because Pt(IV) complexes enter cells by passive diffusion and not via the Pt(II) transporters,^31^ it is intuitively appealing to hypothesize that Pt(IV) prodrugs are not likely to accumulate selectively and efficiently in the DRG and therefore would not induce PIPN.

To test our working hypothesis, we prepared Pt(IV) prodrugs of cisplatin and oxaliplatin with two “innocent” acetato ligands that have no biological activity, [cisPt(OAc)_2_ and oxali(OAc)_2_]. In addition, we prepared the dual-action prodrug, cisPt(Pac)(OH), which in addition to regenerating cisplatin, releases paclitaxel, an anticancer drug that acts on the microtubules (Figure 1C) and is often coadministered with cisplatin in the clinic. We chose paclitaxel because the combination of paclitaxel with platinum drugs shows clinical benefits.^32−35^ Unfortunately, 60–70% of patients receiving paclitaxel suffer from severe neuropathy,^36^ which is dose-limiting for both paclitaxel and docetaxel.^37,38^

This work aims to facilitate the development of efficacious platinum drugs with minimal side effects, including circumventing PIPN with the ultimate goal of improving the patient’s quality of life.

## Results and Discussion

**Figure 2:**
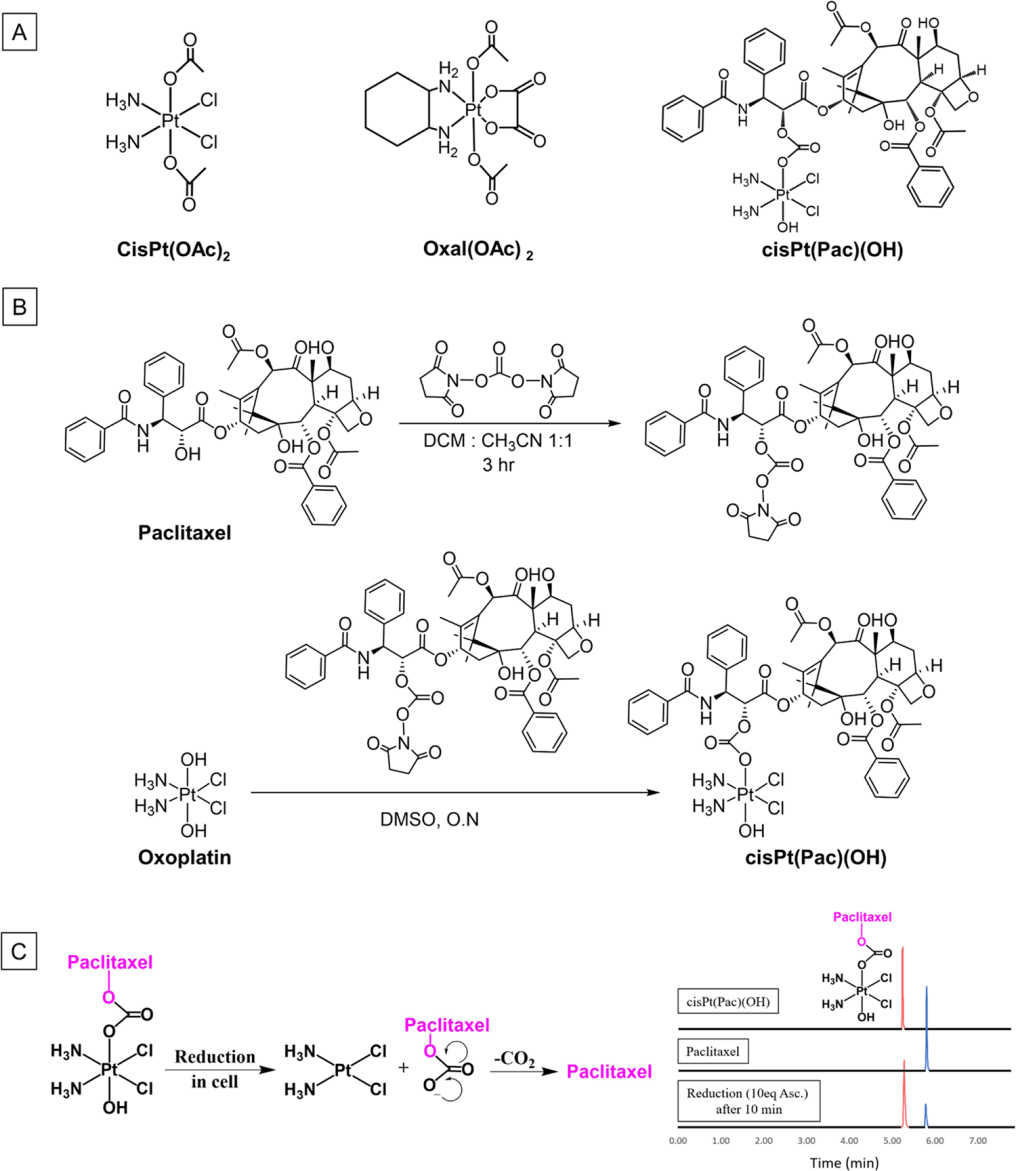
(A) Chemical structures of the Pt(IV) prodrugs used in this study. (B) Synthetic route for the preparation of cisPt(Pac)(OH). (C) Schematic illustration of the reduction of multitargeting prodrug cisPt(Pac)(OH) to release the original, unmodified paclitaxel and HPLC chromatograms of cisPt(Pac)(OH), commercial paclitaxel, and a chromatogram of the reaction mixture following 10 min of incubation with 10 equiv of ascorbate (lower chromatogram) confirming the release of paclitaxel in its native form.

The structures of CisPt(OAc)_2_, oxaliPt(OAc)_2_, cisPt(Pac)(OH), and paclitaxel as well as the synthesis of cisPt(Pac)(OH) are depicted in Figure 2. CisPt(OAc)_2_ and oxaliPt(OAc)_2_ respectively, were prepared by oxidation of the drugs by H_2_O_2_ followed by reaction with acetic anhydride. Because paclitaxel does not possess a carboxylate group, it could not be conjugated directly to the Pt(IV). To conjugate the paclitaxel to the Pt(IV) and to ensure that following reduction the paclitaxel is released in its active form, we tethered the paclitaxel to the axial position of oxoplatin via a self-immolative carbonate linker by first activating the 2′-OH of paclitaxel with *N,N*′-disuccinimidyl carbonate (DSC) and then conjugating it to oxoplatin.^39^

All Pt compounds were purified by HPLC and characterized by ^1^H and ^195^Pt NMR and ESIMS and proved to be >95% pure by HPLC.

Two conditions need to be fulfilled so that Pt(IV) complexes may act as bona fide prodrugs: the prodrugs must be stable in biological fluids and should be activated by reduction inside the cells to release the active form of the drugs to facilitate a quick onset of the anticancer effects.

The stability of the Pt(IV) compounds was measured in RPMI medium, at pH = 7 and 37 °C, by monitoring the peak area of the prodrugs as a function of time. The half-lives of the compounds are provided in Table 1. The order of stability of the compounds is oxali(OAc)_2_ > cisPt(OAc)_2_ > cisPt(Pac)(OH), reflecting the greater stability of Pt(IV) complexes where the axial ligands are conjugated to the Pt(IV) via carboxylates compared with ligands conjugated via carbonates.^40^

**Table 1:** Stability and Reduction Half-Lives of the Pt(IV) Complexes Used in This Study

	*t*_1/2_ RPMI 1640 (h)	*t*_1/2_ 10 equiv Asc (h)
cisPt(OAc)_2_	120	7.8
Oxali(OAc)_2_	742	63
cisPt(Pac)(OH)	35	0.16

To verify that the Pt(IV) prodrugs are activated by reduction, we incubated them with 10 eq. of ascorbic acid at pH = 7 at 37 °C and monitored by HPLC the reduction of the peak of the parent compound as a function of time. The half-lives for the reduction are provided in Table 1. The order of rates of reduction by ascorbate was cisPt(Pac)(OH) ≫ cisPt(OAc)_2_ > oxali(OAc)_2_. This is in agreement with reports that the electron transfer from ascorbate to the Pt(IV) occurs rapidly by inner-sphere electron transfer to OH and Cl ligands but not to amines and carboxylates.^41^ CisPt(Pac)(OH) was rapidly reduced by ascorbate (*t*_1/2_ = 0.16 h) and, following reduction, the free paclitaxel was immediately released, confirming the rapid loss of CO_2_ from the carbonate (Figure 2C).

We tested the *in vitro* potency of cisPt(Pac)(OH) in comparison with cisplatin, paclitaxel, and their 1:1 physical combination against a panel of cancer cell lines, which included human ovarian cancer cells sensitive (A2780) and resistant to cisplatin (A2780cisR), melanoma cells (A375), non-small-cell lung cancer cells (PC9) and murine colon cancer cells (CT26). The results, expressed in terms of IC_50_ values (μM) (IC_50_ – concentration of the compound inhibiting cell growth by 50%), are reported in Table 2.

**Table 2:** IC_50_ Values of Various Chemotherapeutics against Cancer Cells after 72 h Treatment

	IC_50_ values (μM)				
	A2780	A2780cisR	A375	PC9	CT26
cisplatin	1.178 ± 0.13	16.25 ± 3.14	1.91 ± 0.62	5.29 ± 1.75	5.89 ± 1.19
paclitaxel	0.0022 ± 0.001	0.003 ± 0.001	0.014 ± 0.004	0.0045 ± 0.002	0.384 ± 0.109
cisplatin + paclitaxel	0.0033 ± 0.0004	0.0037 ± 0.0008	0.010 ± 0.001	0.0085 ± 0.001	0.282 ± 0.082
cisPt(Pac)(OH)	0.0045 ± 0.002	0.0059 ± 0.0001	0.018 ± 0.003	0.006 ± 0.001	0.406 ± 0.106

As we observed previously with multitargeting Pt(IV) prodrugs,^27,39^ where the axial organic drugs are significantly more potent than the Pt(II) drugs [low nM IC_50_ values for the organic drugs compared with low μM for the Pt(II) drugs], the organic drugs (paclitaxel in this case) determine the cytotoxicity of the prodrug and of the co-treatment of the cells. Cisplatin exhibited low μM IC_50_ values in all cancer cell lines, whereas paclitaxel, the dual-targeting prodrug, cisPt(Pac)(OH), and the 1:1 physical combination of cisplatin and paclitaxel exhibited low nM IC_50_ values, suggesting that the death of the cancer cells was triggered mainly by the paclitaxel, which is released from the prodrug inside the cancer cells.

### *In Vivo* Studies

**Figure 3:**
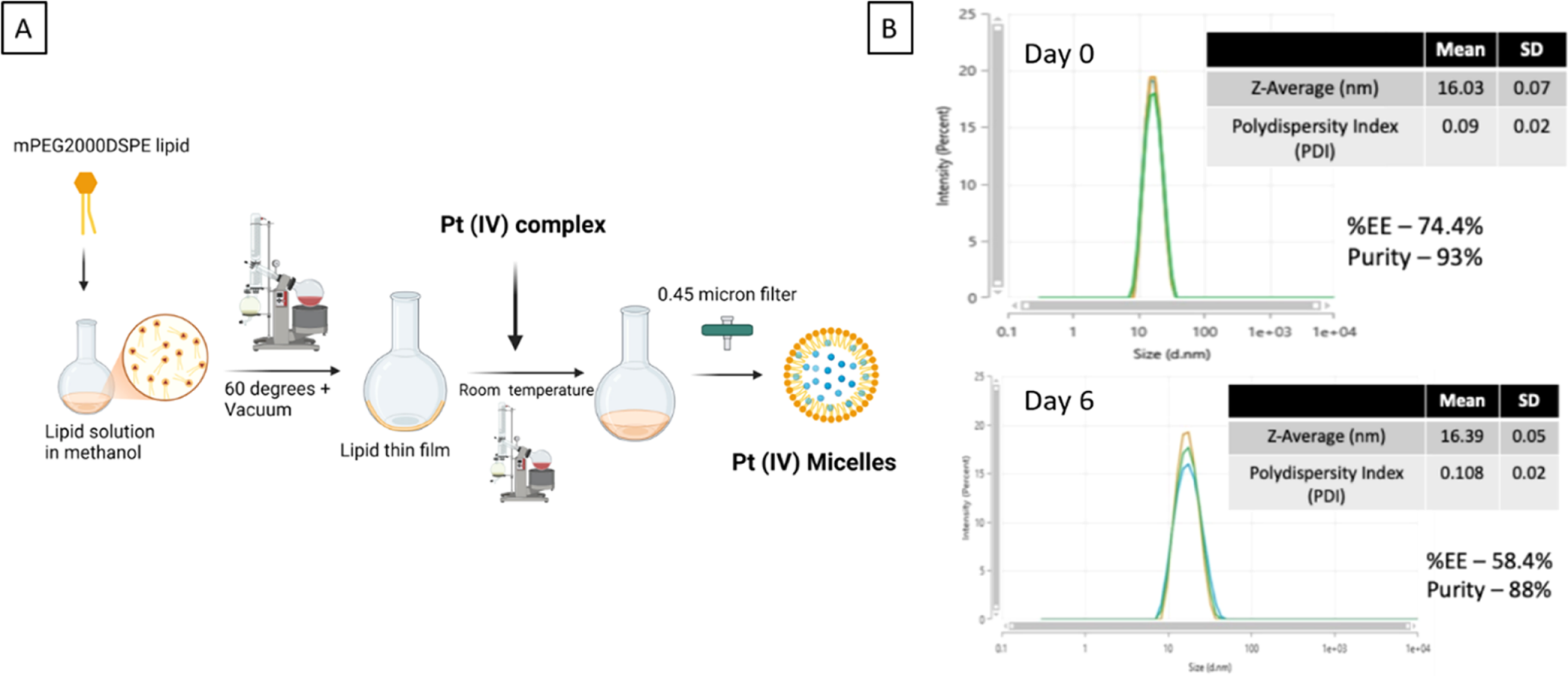
(A) Schematic illustration for the preparation of micellar cisPt(Pac)(OH) via lipid film hydration approach. (B) Stability studies of micellar cisPt(Pac)(OH) determined via Zetasizer for *Z*-average and PDI and HPLC for purity encapsulation efficiency (%EE).

We have recently observed that multitargeting Pt(IV) prodrugs, where the Pt(IV) complex releases a very potent organic anticancer agent, often exhibited good *in vivo* tumor growth inhibition and significantly reduced acute toxicity, lower kidney damage, and decreased hepatotoxicity compared with the individual drugs comprising the Pt(IV) prodrug or with their coadministration as individual entities.^25^ These differences in toxicity profiles between Pt(II) and Pt(IV) complexes prompted us to investigate whether in general, Pt(IV) complexes would induce a lower level of neuropathic pain in mice. In an attempt to further improve efficacy and reduce toxicity, we also encapsulated extremely cytotoxic cisPt(Pac)(OH) into micelles and evaluated its efficacy. Composite micelles containing both a Pt(IV) prodrug of cisplatin and paclitaxel were prepared.^42^ We encapsulated cisPt(Pac)OH complexes inside lipid-based micelles, which enhanced their solubility and stability, with the aim to further facilitate drug accumulation at the diseased areas by taking advantage of the “leaky” vasculature at the tumor site (described as enhanced permeability and retention (EPR) effect).^43,44^ The cisPt(Pac)OH nanoparticles exhibited high encapsulation efficiency (74%) and were stable in suspension for up to 6 days with minimal degradation of the Pt(IV) complex (Figure 3). By protecting the Pt(IV) complex in their inner lipophilic core, these slightly charged nanoparticles, with sizes of about 16 nm, particle concentration of 10^15^ particles/mL and desirable polydispersity index (PDI) of 0.09, were stable for about 1 week upon storage at 4 °C and able to stably circulate in the bloodstream, while preventing the premature recognition by the immune system thanks to their surface decoration with hydrophilic poly(ethylene glycol) (PEG) chains.

### *In Vivo* Inhibition of Tumor Growth

**Figure 4:**
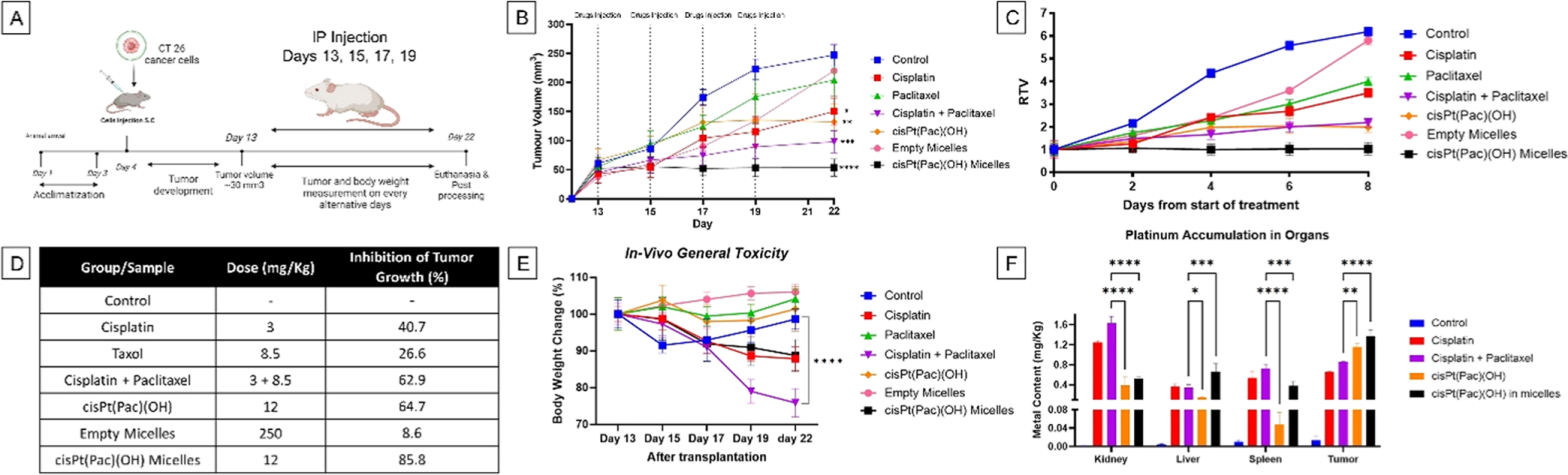
(A) Protocol for the *in vivo* growth inhibition experiments in a CT26 colorectal cancer model upon i.p. injection of the various compounds. (B) Tumor volume changes with time. (C) Relative tumor volumes (Tumor volume day_*x*_/Tumor volume day_13_). (D) Dosages and percentage of solid tumor growth reduction of tested compounds. (E) Percentage body weight changes over time as an indicator for general toxicity. (F) Quantification of the platinum accumulation in the kidney, liver, spleen, and tumor via ICP-MS. *p < 0.05, **p < 0.01, ***p < 0.001, ****p < 0.0001.

We evaluated the efficacy of cisplatin, paclitaxel, 1:1 mixture of cisplatin:paclitaxel, cisPt(Pac)(OH), and micellar cisPt(Pac)(OH) at equimolar concentrations in a murine CT26 colon cancer model. The experimental protocol as well as tumor volume changes and the relative tumor volumes (Tumor volume day_*x*_/Tumor volume day_13_) are depicted in Figure 4A–C. In Figure 4, we also report the doses administered intraperitoneally (ip) and the percentage of tumor growth inhibition (TGI) (Figure 4D), the body weight changes (Figure 4E), and the platinum accumulation in key organs (Figure 4F). It is noteworthy that, under the conditions used, the *in vivo* percent TGI of cisplatin is higher than paclitaxel (41% vs 27%), although its *in vitro* IC_50_ value in the same CT26 cancer cells is ∼15-fold higher (5.89 μM vs 0.384 μM), reinforcing the importance of performing *in vivo* studies.

The results of the *in vivo* efficacy study indicated that, to achieve effective inhibition of tumor growth in the CT26 murine colon cancer model, it was necessary to treat the mice with both platinum and paclitaxel. We utilized three different approaches for the administration of the two drugs: the first was a combination therapy, where each drug is administered separately; the second strategy was to administer a single dual-action prodrug that, following activation in the tumor, releases the two drugs; and the third was to encapsulate the Pt(IV) prodrug in a delivery vehicle (micelles). In the first approach, each drug has its own pharmacokinetic and toxicity profiles and in the second and third approaches, there is only one pharmacokinetic and one toxicity profile. Coadministration of equimolar concentrations of cisplatin + paclitaxel or cisPt(Pac)(OH) were equally efficacious (63 and 65% TGI) though less than the micelle encapsulated prodrug (86% TGI). To make sure that the anticancer effect of encapsulated cisPt(Pac)(OH) derives solely from the prodrug in the delivery vehicle, we subjected mice to high doses of empty micelles (250 mg/kg) resulting in a negligible change in tumor growth inhibition (8.6% TGI) with no decrease in body weight.

The differences in reactivity between reactive cisplatin and more inert Pt(IV) prodrugs can result in different biodistributions that could induce different toxicity profiles. When examining the general toxicity of the compounds, as indicated by body weight loss compared to the control, mice treated with the prodrug, cisPt(Pac)(OH), did not exhibit any substantial weight loss, while those treated with cisplatin, cisplatin + paclitaxel, or cisPt(Pac)(OH) encapsulated in micelles exhibited a significant weight loss. At the end of the experiments, mice were euthanized, and the tumor, spleen, liver, and kidneys were isolated and analyzed in terms of platinum content by ICP-MS (Figure 4F). Interestingly, the platinum levels in the tumor for cisplatin, coadministration of cisplatin and paclitaxel, and cisPt(Pac)(OH) were rather similar, reinforcing the indication that for effective inhibition of the tumor growth, both cisplatin and paclitaxel need to accumulate in the tumor. One of the main side effects of cisplatin is nephrotoxicity that is caused by platinum accumulation in the kidneys.^45^ There were high levels of Pt in the kidneys when the mice were treated with cisplatin alone or in combination with paclitaxel, but much lower levels when treated with cisPt(Pac)(OH) or with cisPt(Pac)(OH) in micelles (Figure 4F). Creatinine measurements and histopathological analyses (Supporting Information), whereby toxic changes to the tubules were associated with focal tubular necrosis in cisplatin samples but not in cisPt(Pac)(OH), suggest lower kidney toxicity for cisPt(Pac)(OH) compared with cisplatin. Higher accumulation in the tumor and lower accumulation in the kidneys for the Pt(IV) complex encapsulated inside micelles were expected, as the micelles increase the bioavailability of the cargo via the EPR effect.^46^

### Chemotherapy-Induced Neuropathic Pain

Platinum-induced neuropathic pain correlates with platination of the DNA in the dorsal root ganglia (DRG).^47^ This reflects the ability of Pt(II) drugs to accumulate in the DRG 10- to 20-fold higher than in other neural tissues.^48^ The greater accumulation in the DRG is attributed to the higher expression levels of the Pt(II) related transporters (OCTs and Ctr1/2) in the DRG.^49,50^ Ravera and co-workers demonstrated *in vitro* that Pt(IV) complexes are not substrates for copper transporters and are internalized through passive diffusion.^31^ Thus, if Pt(IV) prodrugs remain intact *in vivo*, then they might not accumulate efficiently in DRG. To test whether in general, stable octahedral Pt(IV) complexes can circumvent neuropathic pain, we carried out behavioral studies on the potential drug candidate, cisPt(Pac)(OH), and additionally on the inactive Pt(IV) prodrugs of cisplatin and oxaliplatin^51^ with innocent axial acetato ligands that were selected merely as representative Pt(IV) complexes (not as drug candidates). We tested whether they induce mechanical allodynia, heat hyperalgesia, cold allodynia, or spontaneous pain in mice. As reference compounds, we used cisplatin, paclitaxel, cisplatin + paclitaxel, and oxaliplatin, which are known to induce neuropathic pain.

**Figure 5:**
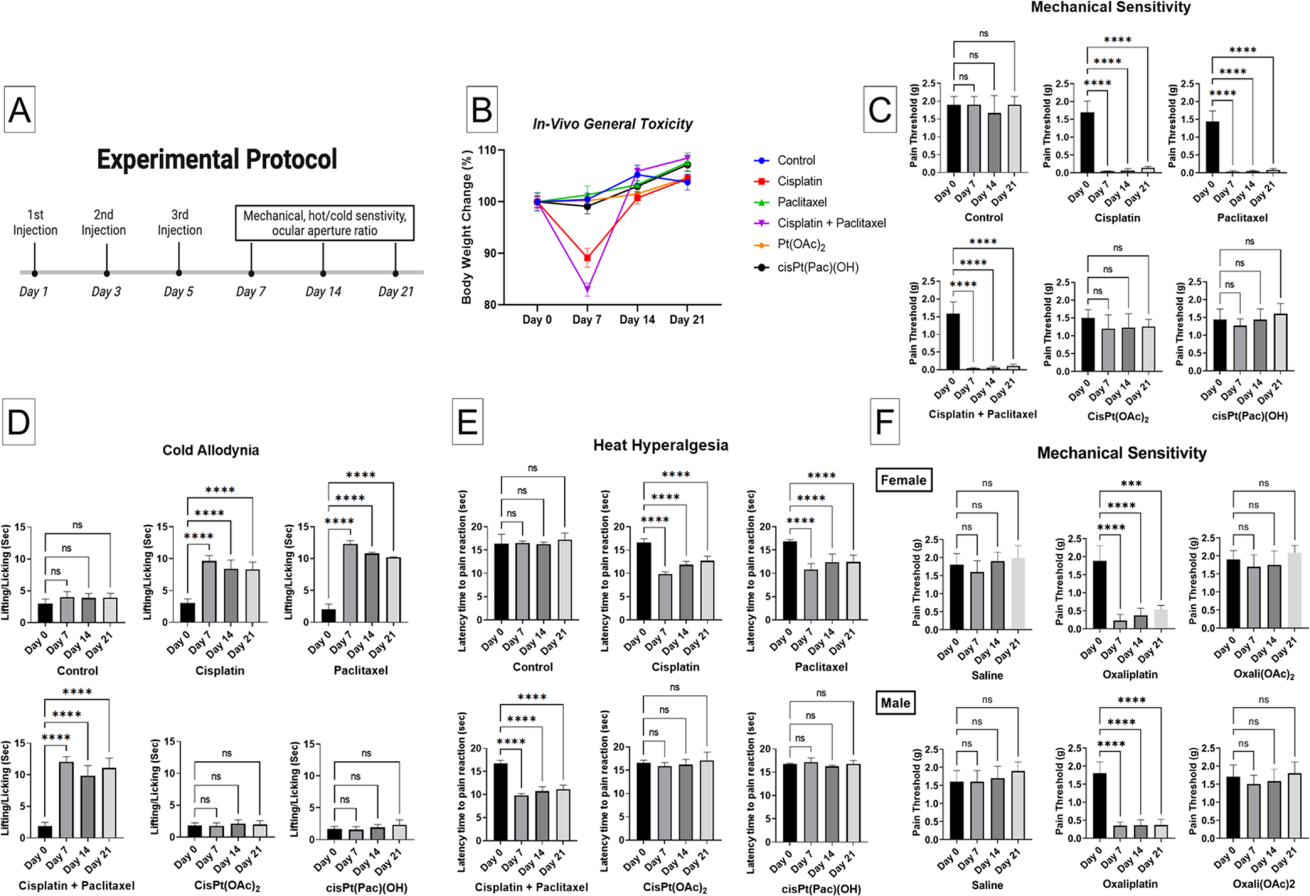
*In vivo* behavioral studies. (A) Experimental protocol (compounds injected i.p). (B) Body weight changes during the experiment. Studies showing changes in (C) mechanical allodynia, via von Frey assay, (D) heat hyperalgesia assessed by the hot plate assay, and (E) cold allodynia examined by the acetone test. (F) Comparison of the mechanical allodynia in male and female mice induced by oxaliplatin. ***p < 0.001, ****p < 0.0001 (one-way ANOVA).

Behavioral studies were conducted using the protocol in Figure 5A. All of the compounds were injected i.p. on days 1, 3, and 5, and the behavioral studies were conducted on days 0, 7, 14, and 21. Body weight loss at equal Pt concentrations indicates that Pt(IV) prodrugs are less toxic than cisplatin or cisplatin+paclitaxel (Figure 5B).

We assessed whether cisPt(OAc)_2_, cisPt(Pac)(OH), and cisplatin induce mechanical allodynia by applying von Frey filaments that exert force, calibrated in grams, to the hind paw of the mice and determining the paw withdrawal threshold. In agreement with previous studies, mice treated with cisplatin or paclitaxel exhibited persistent reduction in the withdrawal threshold compared with the untreated mice (Figure 5C).^52^ Treatment with the 1:1 cisplatin+paclitaxel gave similar results to those obtained with cisplatin or paclitaxel, suggesting that the physical mixture was unable to minimize mechanical allodynia; withdrawal thresholds were in the range of 0.1–0.4 g compared with about 2 g for the untreated mice. Importantly, mice treated with the Pt(IV) prodrugs had significantly higher withdrawal thresholds (1.2–1.4 g), corresponding to a nonstatistically significant reduction of only 24–32% compared with about 95% reduction with cisplatin (*P* < 0.0001 one-way ANOVA).

Similarly, cisPt(OAc)_2_ and cisPt(Pac)(OH) led to significantly reduced cold allodynia (Figure 5D) and thermal hyperalgesia (Figure 5E) compared with cisplatin, paclitaxel, or their 1:1-*co*-administration. Cold allodynia was assessed by cooling the hind paw of the mice with acetone and recording the time (s), during which they displayed aversive behavior. The sensitivity to noxious heat was assessed by placing the mice on a hot plate (54 ± 1 °C) and measuring the latency (s), the time until the mice began licking their paws, shaking, or jumping to avoid the heat.

The data from these three behavioral studies clearly highlight the significant differences between the increased sensitivity to noxious stimuli following treatment with cisplatin or paclitaxel or their coadministration and the lack of neuropathic pain symptoms following the application of the Pt(IV) complexes, cisPt(OAc)_2_ and cisPt(Pac)(OH).

To explore whether Pt(IV) prodrugs in general do not induce neuropathic pain and whether there are differences between male and female mice, we measured withdrawal thresholds to mechanical stimuli following treatment with oxaliplatin, a potent inducer of PN and its Pt(IV) prodrug-oxali(OAc)_2_, in both males and females. We found that, as in the case of cisplatin, the Pt(IV) derivative of oxaliplatin did not induce neuropathic pain. Importantly, we found no differences between the responses of male and female mice (Figure 5F).

**Figure 6:**
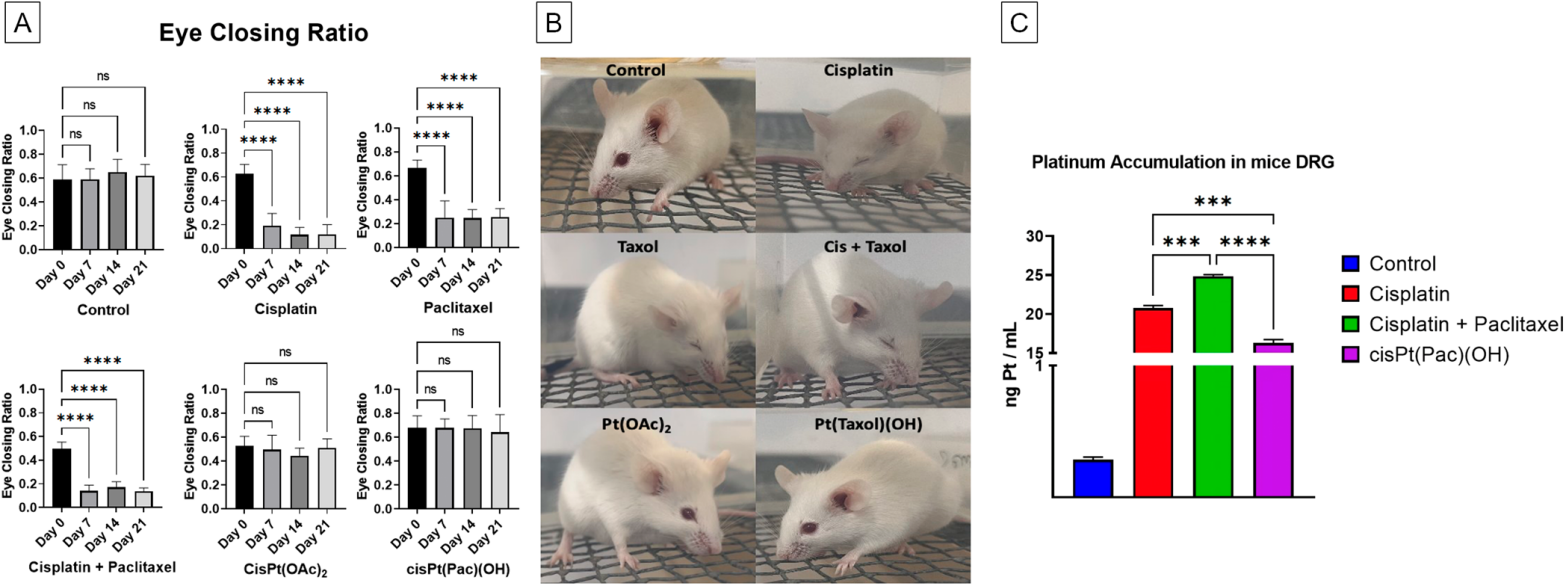
(A) Ongoing pain assessed via eye closing ratio. (B) Example of grimace of awake (not immobilized) mice 21 days post-treatment with the compounds. (C) Quantifications of the platinum accumulation in the DRG on day 7 following injections on days 1, 3, and 5 via ICP-MS. ***p < 0.001, ****p < 0.0001 (one-way ANOVA). (D) Quantifications of the platinum accumulation in the DRG on day 7 following injections on days 1, 3, and 5 via ICP-MS. ***p < 0.001, ****p < 0.0001 (one-way ANOVA).

Finally, we assessed whether the treatment with cisPt(OAc)_2_ or cisPt(Pac)(OH) prevents chemotherapy-induced ongoing pain, by assessing the eye-closing ratio.^53−55^ The eye-closing ratio test was carried out prior to conducting the induced pain experiments. The eye-closing ratio was obtained by dividing the distance between the edges of the upper and lower eyelids by the length of the canthus (horizontal distance across the eye), where a value of 0 was given to completely closed eyes (indicating pain), and a value of 1 was given to fully open eyes (lack of pain). Notably, cisPt(OAc)_2_ and cisPt(Pac)(OH) did not induce any ongoing pain whereas, treatment with cisplatin or paclitaxel led to substantial spontaneous pain (Figure 6).

To explore whether the differences in the behavioral tests between cisplatin, cisplatin + paclitaxel, and cisPt(Pac)(OH) correlate with platinum accumulation in the DRG, we quantified the platinum in the DRG. Platinum levels following treatment with cisPt(Pac)(OH) were lower than after treatment with cisplatin or the co-treatment with cisplatin and paclitaxel (Figure 6).

## Conclusions

The motivation for this study is a need to develop new strategies to prevent neuropathic pain associated with treatment with platinum drugs. Our goal was to explore the possibility of utilizing multitargeting Pt(IV) prodrugs to inhibit tumor growth without inducing severe neuropathic pain. We demonstrated for the first time that Pt(IV) complexes, as simple prodrugs for cisplatin or oxaliplatin, or as multitargeting prodrugs, do not induce neuropathic pain in male or female mice. We suggest that this is likely due in part to the lower accumulation of Pt(IV) in the DRG compared to the Pt(II) drugs. Moreover, the prodrug that combines cisplatin with paclitaxel, both strong inducers of PN, can efficiently inhibit tumor growth *in vivo* without causing body weight loss while reducing accumulation in the kidneys and not inducing neuropathic pain. We have also demonstrated that cisPt(Pac)(OH) can be efficiently encapsulated into micelles forming a stable drug delivery vehicle that enhances the *in vivo* efficacy of the prodrug, We propose that multitargeting Pt(IV) prodrugs have the potential to be more clinically effective than Pt(II) drugs or combination therapy, as they will enable clinicians to use higher dosages while avoiding neuropathic pain and thus significantly improve the quality of life of cancer patients.

## Experimental Section

### Materials and Methods

All chemicals and solvents were procured from authentic commercial sources and used without further purification.

All Pt compounds were characterized by ^1^H NMR, ^13^C NMR, ^195^Pt NMR, and ESI-MS and elemental analysis. Progress of reactions was monitored by analytical HPLC system (Thermo Scientific UltiMate 3000) with a reversed-phase C18 column Phenomenex Kinetex, length 100 mm, internal dia 4.60 mm, particle size 2.6 μm, pore size 100 Å. The purity and retention time (RT) of the synthesized compound reported here were measured with the same analytical HPLC system water/acetonitrile gradient at the flow rate of 1 mL/min. Reaction mixtures were purified on a preparative HPLC system (Thermo Scientific UltimaMate 3000 station) equipped with a reversed-phase C18 column (Phenomenex Luna 250 × 21.2 mm^2^, 10 μm, 100 Å) with a similar type of mobile phase was used with the flow rate of 15 mL/min. UV detection was set at 220 nm in both the HPLC systems. The fractions were combined and lyophilized to get the pure compounds.

All NMR data were collected on a Bruker AVANCE III HD 500 MHz spectrometer. The data were processed by using either MestreNova or Bruker TopSpin 3.6.0 software. ^1^H & ^13^C NMR chemical shifts were referenced with the individual solvent residual peaks of respective NMR solvents used. ^195^Pt NMR chemical shifts were reported with respect to the chemical shift of standard K_2_PtCl_4_ in water at −1624 ppm. Electrospray ionization mass spectra (ESI-MS) were done using a Thermo Scientific triple quadrature mass spectrometer (Quantum Access) by +ve mode electrospray ionization. Elemental analyses reported were performed using a Thermo Scientific FLASH 2000 element analyzer.

### Synthesis

All complexes were synthesized according to reported procedures, purified by HPLC to >95% and characterized by NMR, mass spectrometry, and elemental analysis.

#### Activated Paclitaxel

Paclitaxel (300 mg, 0.35 mmol) was stirred with disuccinylcarbonate (900 mg, 3.51 mmol) and DMAP (86 mg, 0.7 mmol) in a 40 mL 1:1 mixture of DCM & CH_3_CN for 3 h at room temperature. Later, the solvents were evaporated, and the residue was dissolved in DCM (40 mL) and washed with water (3 × 30 mL). The organic layer was then dried using anhydrous sodium sulfate and DCM was evaporated. The activated paclitaxel was purified using HPLC. RT 13.9 min (0–100% linear gradient of acetonitrile in water over 15 min then 3 min constant at 100% acetonitrile, purity >80%). Yield 200 mg (57%).

^1^H NMR (500 MHz, DMSO-*d*_6_) δ: 9.41 (d, 1H, *J* = 8.2 Hz), 8.00 (m, 2H), 7.88 (m, 2H), 7.76 (m, 1H), 7.68 (m, 2H), 7.59 (m, 1H), 7.55 (m, 2H), 7.49 (m, 4H), 7.25–7.22 (m, 1H), 6.29 (s, 1H), 5.87 (m, 1H), 5.64–5.57 (m, 2H), 5.42 (m, 1H), 4.93–4.89 (m, 2H), 4.65 (s, 1H), 4.13–4.06 (m, 1H), 4.04–3.98 (m, 2H), 3.58 (d, 1H, *J* = 7.2 Hz), 2.82 (s, 4H), 2.33–2.27 (m, 1H), 2.25 (s, 3H), 2.11 (s, 3H), 1.84–1.78 (m, 1H), 1.71 (s, 3H), 1.67–1.61 (m, 1H), 1.52–1.48 (m, 4H), 1.03–1.01 (m, 6H); ^13^C NMR (125 MHz, DMSO-*d*_6_) δ: 202.3, 169.7, 169.5, 168.8, 167.8, 166.4, 165.3, 150.9, 138.6, 136.2, 133.9, 133.8, 133.5, 131.7, 129.9, 129.6, 128.9, 128.7, 128.5, 128.3, 127.8, 127.4, 83.6, 80.2, 79.3, 76.6, 75.3, 74.6, 74.5, 71.7, 70.4, 57.4, 53.8, 46.1, 42.9, 36.5, 34.2, 26.3, 25.4, 22.6, 21.3, 20.7, 13.9, 9.8; ESI-MS (*m*/*z*) calculated for [C_52_H_54_N_2_O_18_ + H]^+^: 995.35, found 995.07.

#### ctc-[Pt(NH_3_)_2_(Pac)(OH)Cl_2_]

Oxoplatin, ctc-[Pt(NH_3_)_2_(OH)_2_Cl_2_], (26.7 mg, 0.08 mmol) was stirred overnight at 30 °C with the activated paclitaxel (100 mg, 0.1 mmol) in 3 mL of DMSO. The reaction was monitored through ^195^Pt NMR where we observed a peak around +1050 ppm corresponding to monosubstituted oxoplatin. After completion, the DMSO was extracted with excess diethyl ether to get an off-white precipitate, which was recovered by centrifugation. Redissolving the precipitate in methanol followed by precipitation with excess diethyl ether afforded crude product which was purified by HPLC (RT 12 min, 0–100% linear gradient of acetonitrile in water over 15 min then 3 min constant at 100% acetonitrile, purity >99%). Yield 50 mg (50%).

^1^H NMR (500 MHz, DMSO-*d*_6_) δ: 9.29 (d, 1H, *J* = 8.5 Hz), 7.99 (m, 2H), 7.89 (m, 2H), 7.75 (m, 1H), 7.69 (m, 2H), 7.56 (m, 1H), 7.49 (m, 2H), 7.44–7.39 (m, 4H), 7.14–7.11 (m, 1H), 6.32 (s, 1H), 6.18–5.91 (br, 6H), 5.83 (m, 1H), 5.45–5.40 (m, 2H), 5.13 (m, 1H), 4.91 (m, 1H), 4.87 (m, 1H), 4.53 (s, 1H), 4.12–4.08 (m, 1H), 4.03–3.98 (m, 2H), 3.59 (d, 1H, *J* = 7.2 Hz), 2.54 (s, 1H), 2.36–2.29 (m, 1H), 2.26 (s, 3H), 2.11 (s, 3H), 1.82 (s, 3H), 1.79–1.72 (m, 1H), 1.65 (m, 1H), 1.49 (s, 3H), 1.39–1.35 (m, 1H), 1.02–1.00 (m, 6H); ^13^C NMR (125 MHz, DMSO-*d*_6_) δ: 202.5, 170.4, 169.7, 168.8, 166.0, 165.3, 159.3, 140.3, 137.9, 134.2, 133.5, 132.9, 131.4, 8 129.9, 129.6, 128.7, 128.6, 128.3, 127.9, 127.6, 127.5, 83.6, 80.2, 76.7, 76.2, 75.3, 74.7, 74.5, 70.5, 70.1, 57.3, 54.3, 46.0, 42.9, 36.5, 29.0, 26.3, 22.7, 21.4, 20.7, 14.2, 9.7; ^195^Pt NMR (107.5 MHz, DMSO-*d*_6_) δ: 1051.1; ESI-MS (*m*/*z*) calculated for [C_48_H_57_Cl_2_N_3_O_17_Pt – H]^−^: 1211.26, found 1211.91; Elemental analysis (%) calculated for C_48_H_57_Cl_2_N_3_O_17_Pt (×4.0 H_2_O): C 44.83, H 5.09, N 3.27, found C 44.49, H 4.87, N 3.12.

#### Preparation of Empty Micelles

A PEGylated lipid-based micelle formulation was prepared using 18:0 PEG2000 PE (1,2-distearoyl-*sn*-glycero-3-phosphoethanolamine-*N*-[methoxy(poly(ethylene glycol))-2000]; Avanti Research, Cat. No. 880120P-1g). 50.5 mg of the lipid was dissolved in 2 mL of methanol and transferred to a 10 mL round-bottom flask. The organic solvent was evaporated at 60 °C under a vacuum using the Hei-VAP Core (Heidolph Korea Ltd.) rotary evaporator until a dry lipid film formed. The lipid film was then hydrated with 1 mL of PBS by rotating at room temperature for 30 min without vacuum, followed by overnight incubation. The resulting suspension was filtered through a 0.45 μm filter, and the particle concentration was determined by using a Malvern Zetasizer (ZSU5700, Malvern Panalytical Limited).

#### Preparation of cisPt(Pac)(OH) Micelles

51.6 mg of 18:0 DPSE-PEG2000 PE (Avanti Polar Lipids) and 4 mg of Pt(IV) complex were dissolved in 2 mL of methanol in a round-bottom flask. The organic phase was then completely removed using a rotary evaporator at 60 °C and 50 mbar to form a thin layer of lipid on the bottom of the flask. 1 mL of phosphate-buffered saline (PBS) was then introduced to rehydrate the lipid layer. A 0.2 μm nylon syringe filter was used to remove large aggregates and precipitations after dissolving. The unencapsulated platinum complex was removed by ultracentrifugation (MWCO: 10 kDa) at 2500 g for 10 min at 4 °C twice, and the samples were stored at 4 °C until usage. Micelles were filtered through a 0.2 μm membrane to ensure sterility before *in vivo* studies. The micelles were characterized in terms of hydrodynamic diameter, polydispersity Index (PDI), particle concentrations, and surface charge via dynamic light scattering using the Zetasizer Ultra (Malvern Instruments, Malvern, U.K.).

#### Characterization of Micelles

The hydrodynamic diameter of the micelles was evaluated by dynamic light scattering (DLS) at 25 °C using a Zetasizer and resulted in a *Z*-average of 16.03 nm and polydispersity index (PDI) of 0.09 for cisPt(Pac)(OH) as an average of three separate measurements. The ζ-potential recorded was −7.5 mV. The particle concentration measured for Pt(Taxol)(OH) was 1 × 10^15^ particles/mL.

#### Loading Encapsulation Efficiency and Stability

Determination of drug encapsulation was performed by HPLC using a calibration curve through gradient concentrations of the Pt(IV) compound. In order to break up the micelles, 50% volume of MeOH was added to the HPLC vial with AUC and degradation percentage taken into account. The stability of the formulation was assessed upon storage at 4 °C for a week (6 days) in terms of micelles’ size and PDI and Pt(IV) drug loading.

### *In Vitro* Studies

#### Reduction Studies

All reduction studies of reported Pt(IV) complexes were performed in the presence of 10 equiv of ascorbic acid at pH 7.4 and 37 °C in the dark and were monitored by analytical HPLC operating with 0–100% linear gradient of water (or 0.1% TFA in water) to acetonitrile as mobile phase over 5.84 min + 2 min 100% acetonitrile. Samples were dissolved in accordance with their solubility in 1:1 MeOH/phosphate buffer (100 mM, pH 7.0) or only MeOH in which pH was adjusted to pH 7.0 using sodium hydroxide.

The half-lives (*t*_1/2_) of all compounds were obtained after linear fitting of ln(*A_t_*/*A*_0_) vs time (*t*) by considering a pseudo-first-order rate equation (*A_t_* = *A*_0_*e*^–*kt*^), where *A*_0_ and *A_t_* are the integrated areas of HPLC peaks of the respective complexes at *t* = 0 and at time *t*, respectively, and *k* (slope) is the rate constant. Then, *t*_1/2_ was calculated by utilizing the equation *t*_1/2_ = 0.693/*k*.

#### Stability Studies

Pt(IV) complexes were dissolved in 1% DMSO and RPMI medium at pH 7.4, and kept at 37 °C. Stability half-lives, *t*_1/2_, were calculated by linear fitting of ln(*A_t_*/*A*_0_) vs time (*t*), where *A_t_* and *A*_0_ are the integrated peak areas by HPLC at *t* = 0 and at time (*t*).

#### Antiproliferative Assay in Tumorigenic Cells

A375 melanoma cells (5000 cells/well), A2780 ovarian carcinoma cells (5000 cells/well), A2780cisR cisplatin-resistant ovarian carcinoma cells (7000 cells/well), PC9 small lung cell carcinoma cells (5000 cells/well), and CT26 WT murine colon carcinoma cells (5000 cells/well) were seeded in flat-bottom 96-well plates and incubated at 37 °C/5% CO_2_ for 24 h. Stock solutions of the compounds in DMSO [cis(OAc)_2_, Oxali(OAc)_2_, cisPt(Pac)(OH)] or in cell culture medium (Cisplatin & Oxaliplatin) were freshly prepared and diluted with the respective cell culture medium to graded concentrations (final concentration of DMSO: 0.1% v/v). After 72 h of exposure, the cell biomass was determined by 3-(4,5-dimethylthiazol-2-yl)-2,5-diphenyl tetrazolium bromide (MTT) (Sigma-Aldrich) staining, and the absorptions were calculated as the delta of two measurements of the same well at wavelengths 570 and 690 nm. IC50 values were determined as the concentration that caused 50% inhibition of cell proliferation compared to an untreated control. The results were calculated as the mean values of three independent experiments.

#### Cell Culture Maintenance

A2780 human ovarian carcinoma cells and A2780cisR cisplatin-resistant ovarian carcinoma cells were cultured in RPMI 1640 (no glutamine) supplemented with heat-inactivated fetal bovine serum (South American origin) (10% v/v), gentamycin sulfate solution (1% v/v), and l-glutamine solution 2 mM (1% v/v). PC9 human non-small-cell lung carcinoma cells, A375 human malignant melanoma cells, and CT26 WT murine colon carcinoma cells were maintained in DMEM (high glucose, no glutamine/sodium pyruvate) supplemented with l-glutamine solution (4 mM) (2% v/v), 10% (v/v) heat-inactivated fetal bovine serum (South American origin, European grade), and 1% (v/v) penicillin–streptomycin solution (containing penicillin G sodium salt: 10 000 units/mL and streptomycin sulfate: 10 mg/mL) and were passaged twice a week. Once a week, a solution of cisplatin in RPMI medium (end concentration: 1 μM) was added to A2780cisR cells to maintain their resistance. All reagents were purchased from Biological Industries. A375, PC9, A2780, and A2780cisR cells were obtained from the American Type Culture Collection (ATCC, Rockville, MD), and CT26 WT was obtained from Prof. Galia Blum lab.

### *In Vivo* Studies

All *in vivo* efficacy studies were carried out at the National University of Singapore (NUS) in accordance with Responsible Care and Use of Laboratory Animals (RCULA) guidelines via two approved Institutional Animal Care & Use Committee (IACUC) protocol:

- National University of Singapore–Protocol Number: R2021-0034
  . All *in vivo* behavioral studies were carried out at The Hebrew University of Jerusalem (HUJI) in accordance with Responsible Care and Use of Laboratory Animals (RCULA) guidelines via two approved Institutional Animal Care & Use Committee (IACUC) protocol.
- The Hebrew University of Jerusalem–Protocol Number: MD-23-17349-4. Each *in vivo* experiment contained six mice per group: tumor inhibition, behavioral studies (von Frey, hot plate, acetone evaporation, spontaneous ocular discomfort), and DRG accumulation.

### *In Vivo* Efficacy Studies

Four-week-old female BALB/cAnNTac mice weighing 16–18 g were purchased from InVivos (Singapore). The animals were housed in Comparative Medicine (CM)-holding units at the National University of Singapore in a pathogen-free environment at constant temperature in a 12/12 h light/dark cycle environment. The mice were acclimatized for 3 days and given access to food and water ad libitum. The mouse colon carcinoma cells CT26 (1 × 10^5^ cells in 100 μL of sterile PBS) were subcutaneously injected into the right flank of the mice. The administration of tested compounds was started when tumors became palpable (volume of ∼30 mm^3^) on day 13. Subsequently, the animals were separated into 5 groups (*n* = 6 each) and injected i.p. with different compounds every other day up to 4 doses. Control mice were injected ip with 100 μL of sterile saline. The animals were controlled for pain and/or distress development, and the body weight and tumor size were closely monitored. All animals were alert and responsive during the whole experiment. The mice were euthanized when the diameter of the tumor reached 1.5 cm or upon development of ulceration. The tumor and organs (liver, kidney, and spleen) were collected after the mice.

### Histopathological Evaluation of Kidney (H&E Staining)

All of the kidney (left kidney) samples were flash-frozen immediately after weighing for cryosections for the histopathological evaluation using H&E staining technique. The tissues were embedded in the OCT compound and 10 μm thick sections were cut and placed onto glass slides using the Leica CM3050 S Cryostat. The slides were dried in a desiccator for approximately 1 h before rinsing in water. Sections are ready for staining immediately after rinse. The slides were stained with H&E (Hematoxylin and eosin) using Leica Autostainer XL. Then, the slides were dehydrated through ascending series of ethanol to xylene before being coverslipped using Leica Coverslipper CV5030. The slides were imaged using the LABOMED Opti CX biological microscope. At least two sections of each kidney sample were stained and 5–10 images were taken for each section under 10× and 40× magnifications. The images were analyzed for histopathological changes by two pathologists in a blinded manner.

### Pain Behavioral Studies

Von Frey, hot plate, acetone evaporation, and ocular aperture ratio studies were carried out in Balb/c mice according to reported methods.^3^ Experiment protocol consisted of a series of three injections (days 1, 3, 5) of saline (control), cisplatin (3 mg/kg), oxaliplatin (4 mg/kg), paclitaxel (8.5 mg/kg), cisplatin & paclitaxel mixture (3 + 8.5 mg/kg), and platinum(IV) complexes dosages were adjusted to Pt amount—Cis(OAc)_2_ (4.16 mg/kg), Oxali(OAc)_2_ (5.19 mg/kg), and Cis(Paclitaxel)(OH) (12 mg/kg). All solutions were freshly prepared and animals were weighed before each injection; Cisplatin, Oxaliplatin, Cis(OAc)_2_, and Oxali(OAc)_2_ were dissolved in saline. Paclitaxel, Cisplatin + Paclitaxel mixture, and Cis(Paclitaxel)(OH) were dissolved in a 1:1 mixture of EtOH and cremophor EL (Sigma-Aldrich) and further diluted in saline (1:3). Final injection volume was 150 μL. Afterward, tests were carried out 1, 2, and 3 weeks post first injection.

### Von Frey Assay

8-week-old Balb/c mice were purchased from Envigo (Israel). Pain thresholds were assessed by observing withdrawal responses to mechanical stimulation of the plantar skin by using von Frey filaments. The mice were placed on a grid and allowed to accustom to the new environment for at least half an hour. The hind paw was touched vertically on the plantar surface with the tip of the filament. Once the fiber bends, the force applied is the maximum, making the repetitions reproducible. Hairs calibrated for forces between 0.008 and 2 g were applied 10 times each in ascending order of force at 5 s intervals to both hind paws. Sharp and quick leg withdrawal or immediate jumping in response to a touch with the hairs was considered as a withdrawal response. Paw movements associated with locomotion were not counted as the withdrawal response. The threshold of withdrawal response was defined when 6 out of 10 applications elicited pain responses. each group contained six animals.

### Hot Plate Test

Unrestrained mouse was placed on a metal surface that was maintained at a constant temperature (54 ± 1 °C) and the response latency, which is the time taken to observe a nocifensive behavior, was recorded. These behaviors include fore paw withdrawal or licking, hind paw withdrawal or licking, stamping, leaning posture, and jumping. each group contained six animals. If no nocifensive behaviors were observed, the animals were removed from the hot plate after 30 s to prevent tissue damage.

### Acetone Evaporation Test

The test was carried out on a mesh floor, and acetone was dabbed on the plantar surface of the hind paw. Sensitivity to cold was recorded by the duration of nocifensive responses such as brisk withdrawal, flick of the paw, repeated flicking of the paw, repeated flicking of the hind paw, and licking of the paw. each group contained six animals.

### Eye Closure Ratio

The raw data for the ongoing pain was obtained by taking 3–5 photographs of each of the awake (not immobilized) mice and used such that the position of the eyelids in relation to the eye was clearly visible. The data were analyzed in a blind fashion by using the imaging software, ImageJ, to measure the length between the internal and external canthus (*x*) and the distance between the edge of the upper and lower eyelids (*y*). Finally, the ratio of *y*/*x* was calculated, and a value between 0 and 1 was obtained for each photo, where 0 is equivalent to the eye completely closed and 1 is equivalent to the eye completely open. The values obtained were an average of the values of the six animals in each group.

### DRG Isolation

Saline (150 μL), cisplatin (3 mg/kg), cisplatin + paclitaxel (3 + 8.5 mg/kg), and cisPt(Taxol)(OH) (12 mg/kg) were injected into female BALB/c OlaHsd mice (six mice per group)–8 weeks of age at days 1, 3, and 5. Afterward, on days 7–10, animals from each group were anesthetized using isoflurane and decapitated. Thereafter, DRG were removed and placed in DDW at room temperature.

### Platinum Accumulation in Organs

Samples were weighed and mineralized with highly pure 70% nitric acid (Pt: ≤0.01 μg kg^–1^, TraceSELECT Ultra, Sigma Chemical Co.) for 3 h. Afterward, solutions were completely dried at 110 °C and 1000 μL of 1% HNO_3_ was added. The platinum content of each sample was measured by ICP-MS. The calibration curve was obtained using known concentrations of standard solutions purchased from Sigma Chemical Co.

## Data Availability

The authors declare that data supporting the findings of this study are available within the paper and its Supporting Information. The data is available from the authors upon request. Source data are provided in this paper.

## Supporting Information Available

Additional description of the synthetic approach as well as the characterization of the cisPt(Pac)(OH) by NMR and ESIMS and purity assessment by HPLC; creatinine levels in serum and the H&E staining of kidnaps in CT26 tumor model (PDF)
Molecular formula strings (CSV)

## Abbreviations Used

ASCO: American Society of Clinical Oncology
CIPN: chemotherapy-induced peripheral neuropathy
DCM: dichloromethane
DMAP: 4-dimethylaminopyridine
DMSO: dimethyl sulfoxide
DRG: dorsal root ganglia
DSC: *N*,*N*′-disuccinimidyl carbonate
EPR: enhanced permeability and retention effect
ESIMS: electron spray ionization mass spectroscopy
HPLC: high-pressure liquid chromatography
ICP-MS: inductively coupled plasma mass spectrometry
NMR: nuclear magnetic resonance
PBS: phosphate-buffered saline
PEG: poly(ethylene glycol)
PIPN: platinum-induced peripheral neuropathy
PN: peripheral neuropathy
TGI: tumor growth inhibition
