# Supplementary material for: Multitargeting Pt(IV) Derivatives of Cisplatin or Oxaliplatin Inhibit Tumor Growth in Mice without Inducing Neuropathic Pain
Source: J Med Chem. 2025 Jan 8;68(2):1608–18. doi: 10.1021/acs.jmedchem.4c02263 (PMC11770746; doi:10.1021/acs.jmedchem.4c02263)
Supplement: Supplementary file 1 — jm4c02263_si_001.pdf [file jm4c02263_si_001.pdf]

Electronic Supplementary Information (ESI)

Multi-targeting Pt(IV) derivatives of cisplatin or oxaliplatin inhibit tumor growth in mice without inducing neuropathic pain

Tomer Babu[1], Ram Pravin Kumar Muthuramalingam[2], Wei Heng Chng[2], Nicolette Yau[2], Sourav Acharya[1], Nurit Engelmayer[3,4], Rachel Feldman-Goriachnik[5], Shaya Lev[3,4], Giorgia Pastorin*[2], Alexander Binshtok*[3,4], Menachem Hanani*[5] and Dan Gibson*[1]

[1] Institute for Drug Research, School of Pharmacy, The Hebrew University of Jerusalem, Jerusalem-9112102, Israel

[2] Department of Pharmacy and Pharmaceutical Sciences, Faculty of Science, National University of Singapore, Singapore, 117544, Singapore

[3]Department of Medical Neurobiology; Institute for Medical Research Israel-Canada, The Hebrew University-Hadassah School of Medicine, Jerusalem, 91120 Israel

[4]The Edmond and Lily Safra Center for Brain Sciences, The Hebrew University of Jerusalem, 9190401 Israel

[5] Laboratory of Experimental Surgery, Hadassah-Hebrew University Medical Center, Mount Scopus, Jerusalem 91240, Israel, and Faculty of Medicine, Hebrew University of Jerusalem, Jerusalem, Israel.

*Corresponding authors E-mail: phapg@nus.edu.sg, alexander.binshtok@mail.huji.ac.il, hananim@mail.huji.ac.il and dang@ekmd.huji.ac.il

Table of Contents

Figure S1 Synthetic Approach for Pt(IV) complexes bearing acetate axial ligands 3

Figure S2 ^{1}H NMR of CisPt(Pac)(OH) in DMSO-d_6 4

Figure S3 ^{195}Pt NMR of CisPt(Pac)(OH) in DMSO-d_6 5

Figure S4 ESI-MS (-ve) of CisPt(Pac)(OH) 6

Figure S5 HPLC Chromatogram of CisPt(Pac)(OH) – 0-100% acetonitrile in 5.84 min + 2 min constant 100% acetonitrile. 7

Figure S6 Creatinine levels in serum 8

Figure S7 H&E Staining of Kidney in CT26 Tumor Model 11

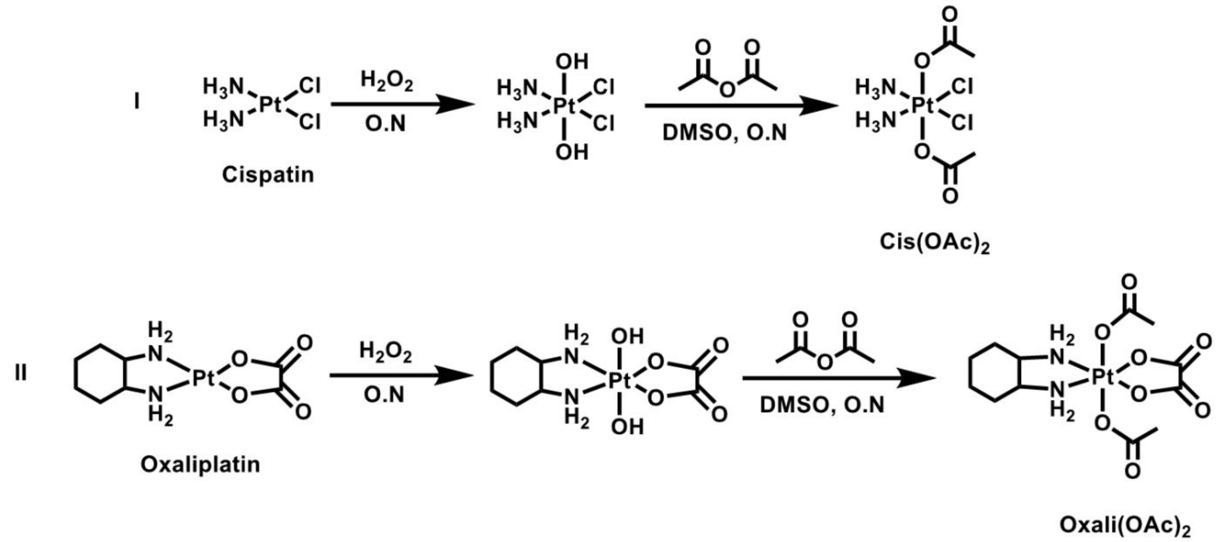

Figure S1 Synthetic Approach for Pt(IV) complexes bearing acetate axial ligands

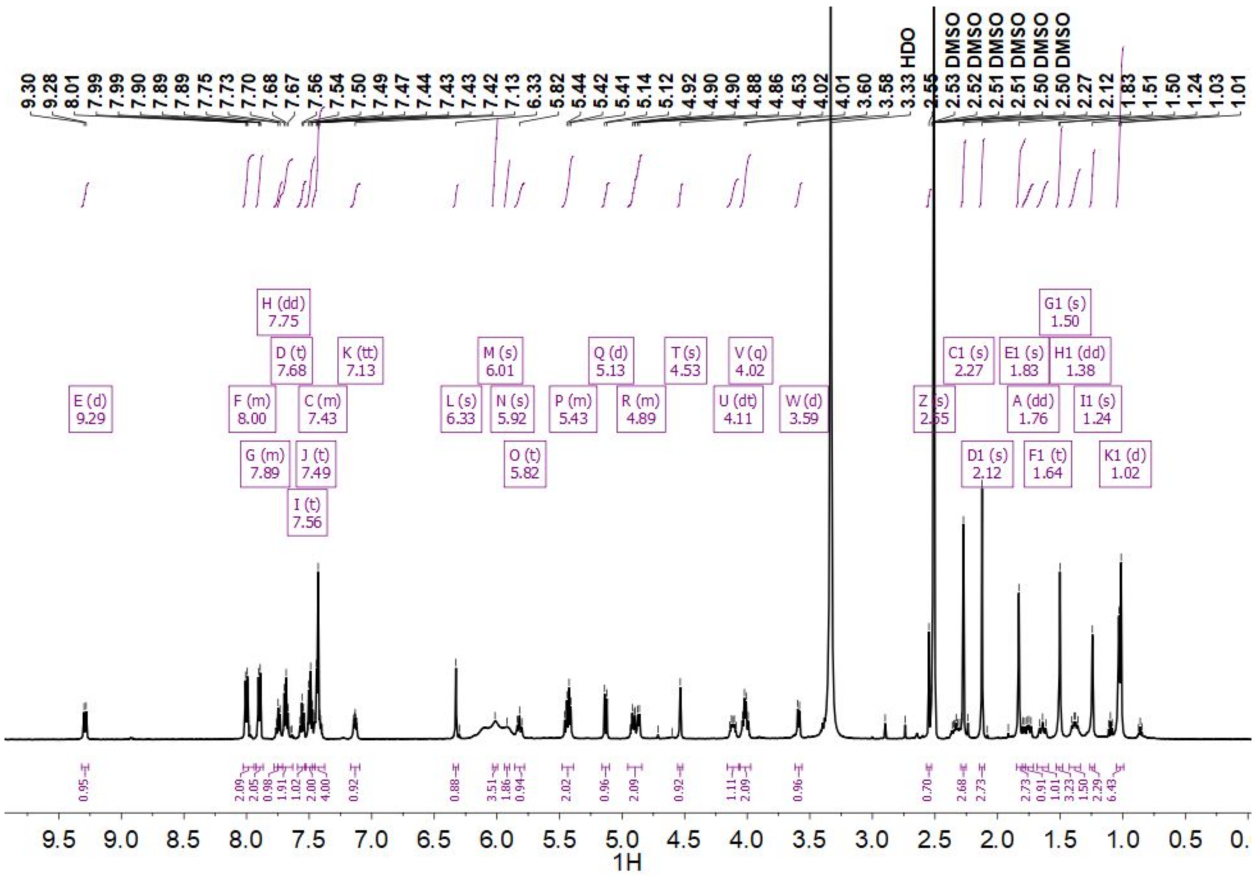

Figure S2 ^{1}H NMR of CisPt(Pac)(OH) in DMSO-d_6

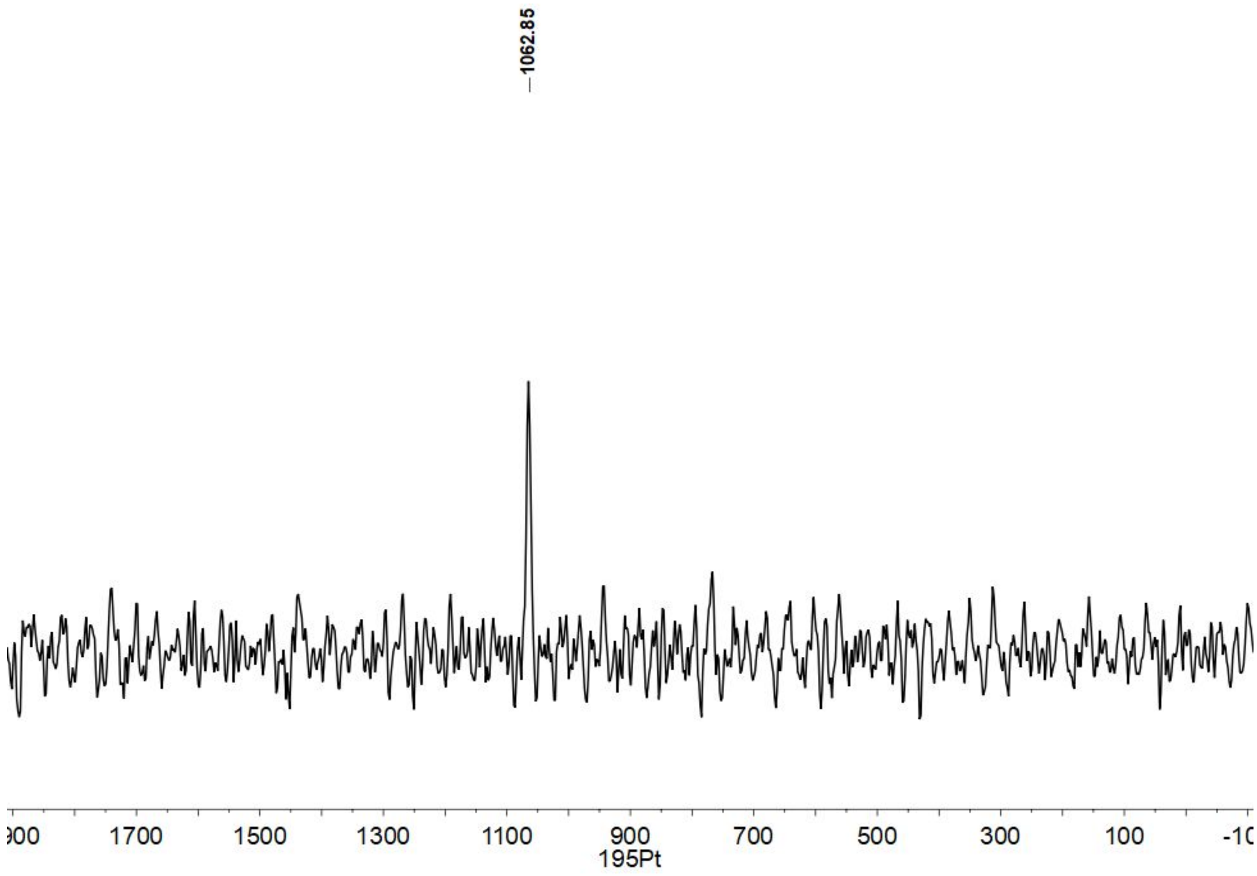

Figure S3 ^{195}Pt NMR of CisPt(Pac)(OH) in DMSO-d$_6$

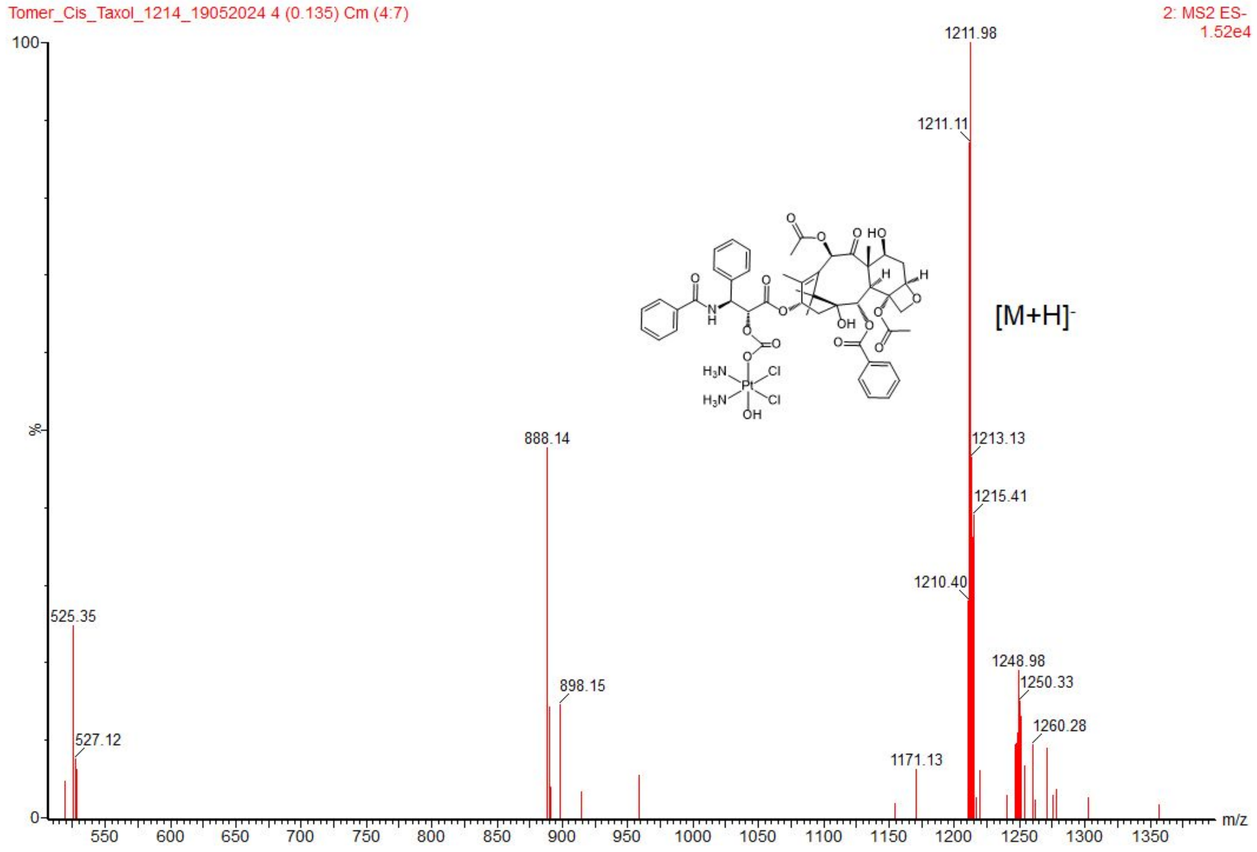

Figure S4 ESI-MS (-ve) of CisPt(Pac)(OH)

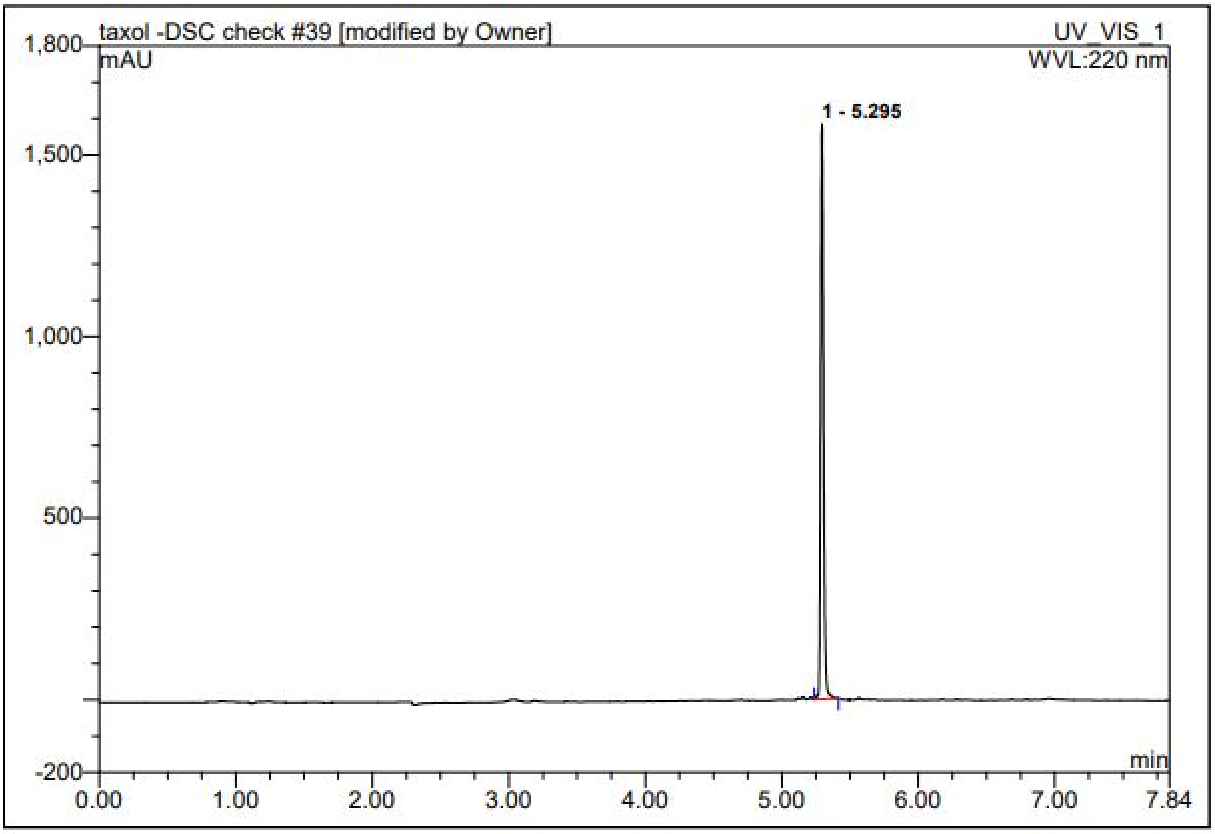

Figure S5 HPLC Chromatogram of CisPt(Pac)(OH) – 0-100% acetonitrile in 5.84 min + 2 min constant 100% acetonitrile.

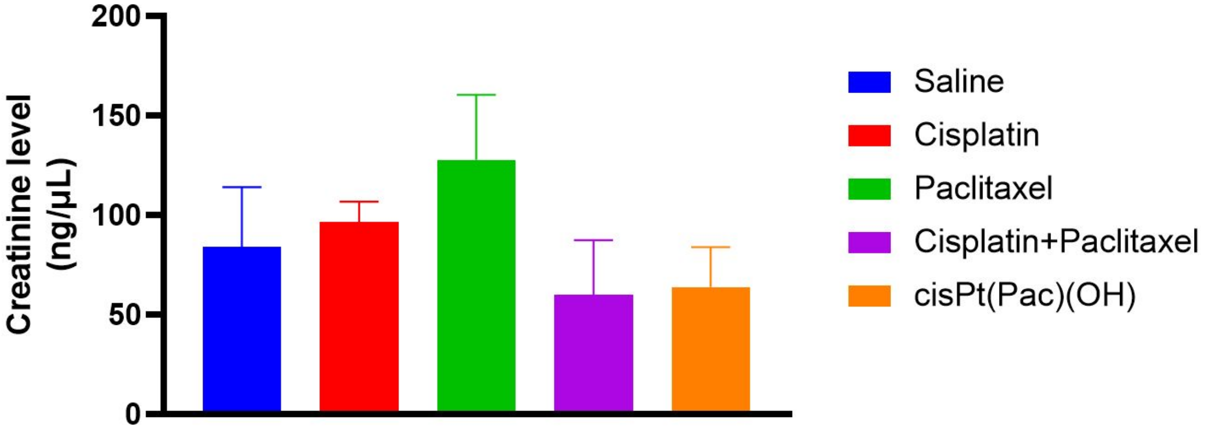

Figure S6 Creatinine levels in serum

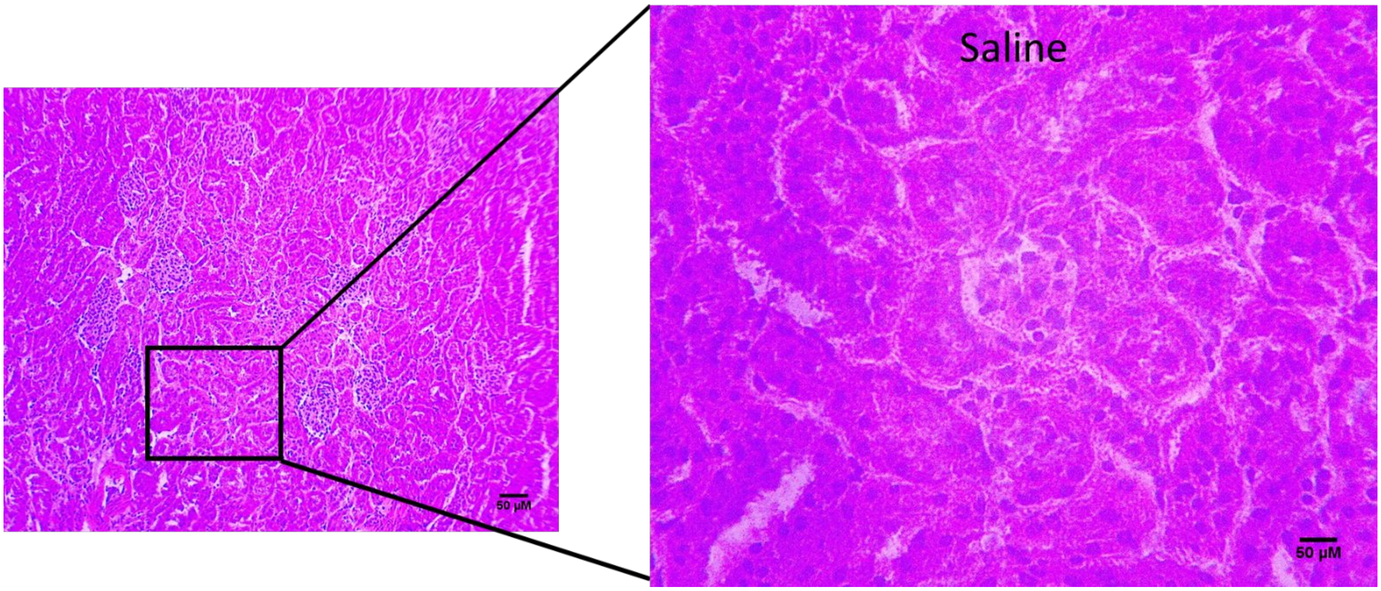

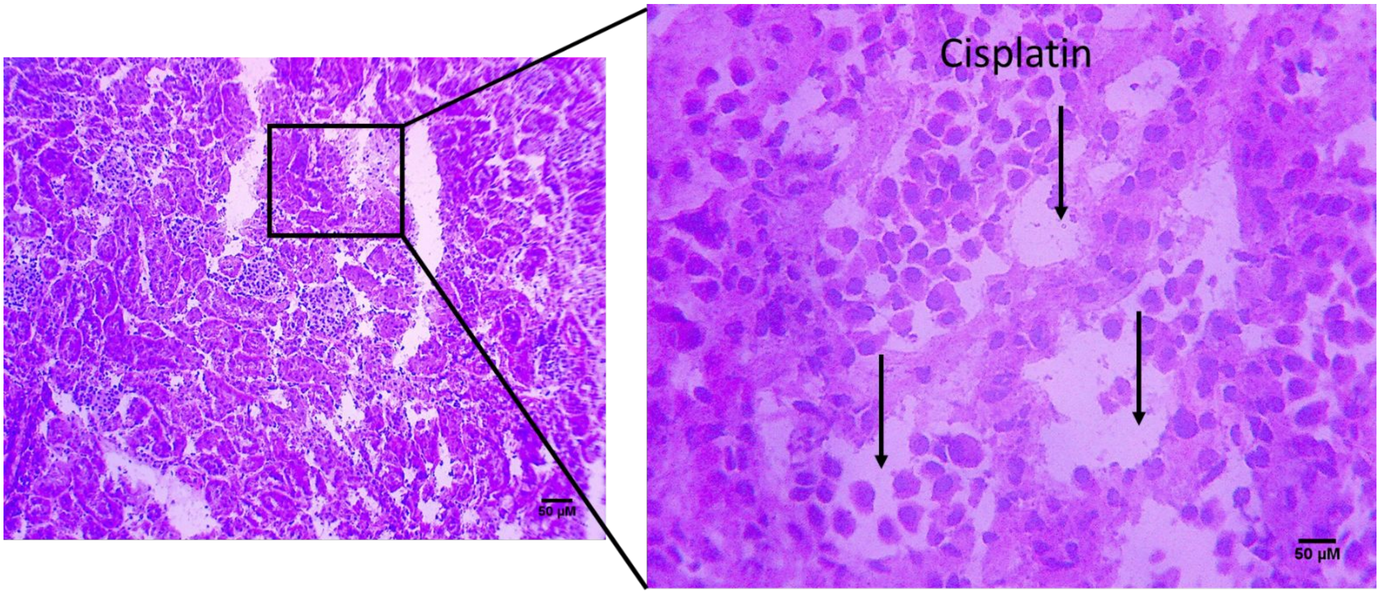
Cisplatin
50 µM
50 µM

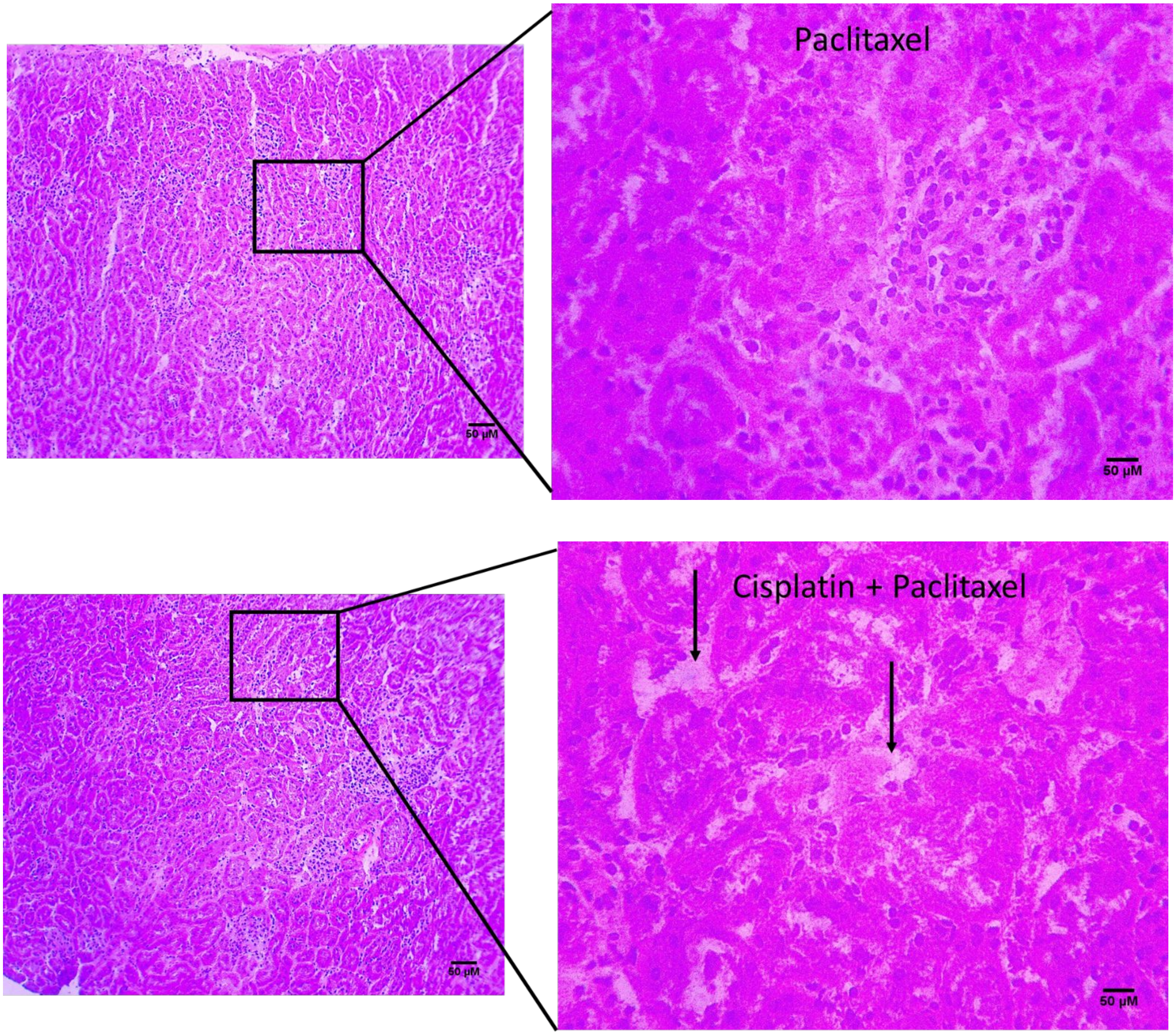
Paclitaxel
50 μM
50 μM
Cisplatin + Paclitaxel
50 μM
50 μM

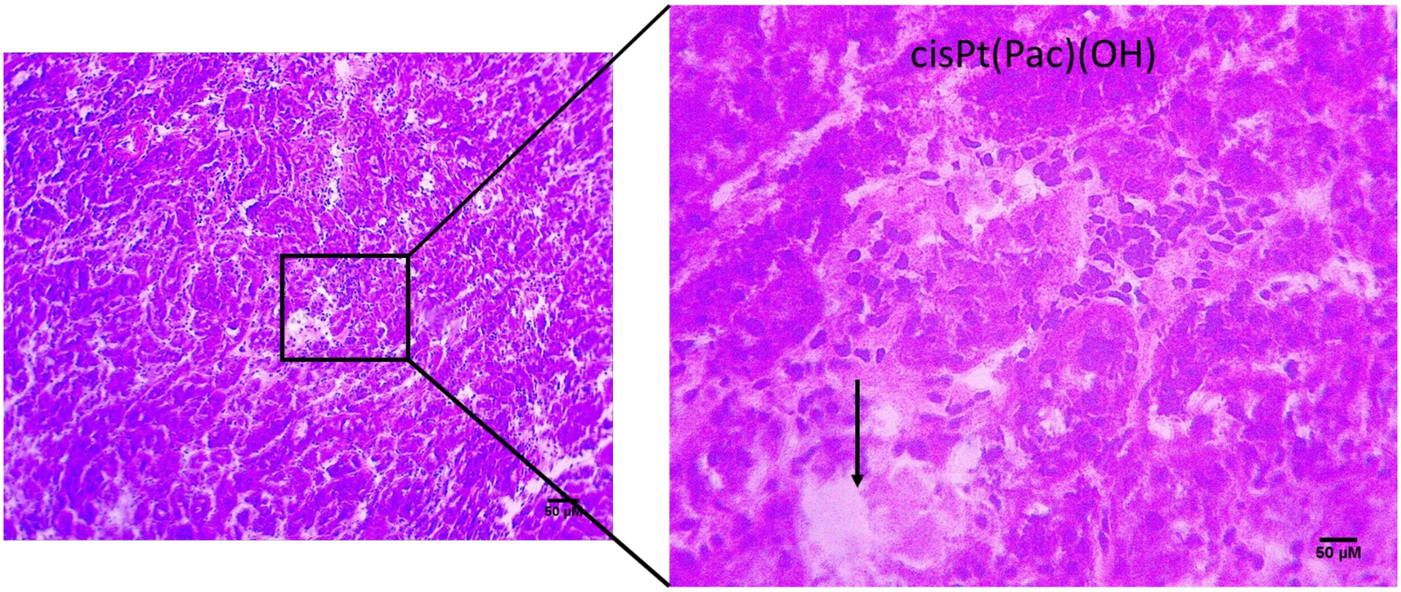

Figure S7 H&E Staining of Kidney in CT26 Tumor Model
